# Mechanistic insights into PVC microplastic adsorption on montmorillonite: A first-principles approach toward pollution control

**DOI:** 10.1007/s11356-026-37449-w

**Published:** 2026-02-05

**Authors:** Hafiz Muhammad Umer Aslam, Achintya Bezbaruah, Dmitri Kilin

**Affiliations:** 1 Civil, Construction and Environmental Engineering, North Dakota State University, Fargo, ND 58102 USA; 2 Environmental and Conservation Sciences, North Dakota State University, Fargo, ND 58102 USA; 3 Chemistry and Biochemistry, North Dakota State University, Fargo, ND 58102 USA; 4 Department of Chemistry, School of Science, University of Management and Technology, Lahore, 54770 Pakistan

**Keywords:** Plastic pollution, DFT, MD, PDOS, Vinyl chloride, MMT, RMSD

## Abstract

Plastic pollution has emerged as a pervasive environmental threat, with polyvinyl chloride (PVC) being a persistent polymer that can degrade into smaller fragments. These particles contaminate aquatic environments, accumulate in biota, and pose serious ecological and health risks. This study used computational methods to investigate the adsorption of vinyl chloride (VC), a PVC oligomer, onto montmorillonite (MMT). Vienna Ab initio Simulation Package was used to perform Density Functional Theory calculations. The interaction between VC and MMT was assessed through binding energy, density of states (DOS), projected DOS, and charge analysis. A negative binding energy (− 0.62 eV) confirmed favorable adsorption. The reduced HOMO–LUMO gap in the VC–MMT hybrid indicated electronic interactions. The orbital-resolved projected density of states (PDOS) showed overlap between the O 2p orbitals of MMT and the H 1 s orbitals of VC. Bader charge analysis revealed negligible charge transfer to the VC molecule upon adsorption, while charge density difference showed localized electron redistribution at the VC–MMT interface. These results indicate a noncovalent interaction without the formation of shared charge density, consistent with polarization-driven physisorption. Molecular dynamics simulations supported these interactions, showing that the VC molecule remained associated with the MMT surface through noncovalent forces. Root mean square deviation (RMSD) confirmed that the VC–MMT structure remained stable throughout the simulation, while the interaction energy exhibited stable fluctuations over time. These findings suggest that MMT holds potential as an effective sink for PVC microplastics through stable, non-covalent surface retention, thereby reducing their dispersion in environmental matrices.

**Graphical Abstract:**

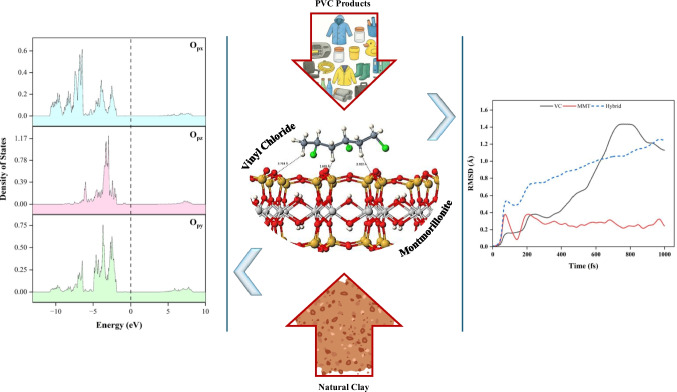

**Supplementary Information:**

The online version contains supplementary material available at 10.1007/s11356-026-37449-w.

## Introduction

Plastics are organic polymers produced synthetically and contain repeating atoms of carbon. They are abundantly used in different industries due to their versatility, low cost, durability, and lightweight nature. The growing dependence on plastics has driven their large-scale production, leading to massive waste generation. Their limited recycling and improper disposal led to the accumulation of waste in different environmental matrices (Khan and Iqbal 2023; Kolganov et al. 2023; Landebrit et al. 2025; Motyl and Fischer 2025). They are not degradable under normal conditions, which further worsens the issue. Additional concerns arise when larger plastics break down into smaller fragments and form microplastics (MPs) (0.1 µm–5 mm) and Nanoplastics (NPs) (< 0.1 μm) (Chen et al. 2022; Jiang et al. 2023; Li et al. 2016; Liu and Wen 2024). Such fragmentation increases their mobility and persistence in aquatic systems (Koelmans et al. 2019; Pannetier et al. 2020; Yang et al. 2021). Studies have also identified these tiny particles in the terrestrial ecosystem and in the atmosphere, which showed their presence beyond aquatic environments and highlights the interconnected nature of plastic pollution (Fang et al. 2024; Taudul et al. 2024).

There are various forms of plastics in the environment, such as fibers, fragments, beads, and sheets (Zhang et al. 2020). The common types of plastic polymers are polyvinyl chloride (PVC), polyethylene (PE), and polystyrene (PS) (Burns and Boxall 2018; Sharmin et al. 2024; Taudul et al. 2024). Polyvinyl chloride (PVC) is widely used in construction, automotive, healthcare, and electronic applications, particularly in products such as pipes, cables, films, and packaging materials. Polyethylene (PE) is commonly used in packaging, plastic bags, films, bottles, and agricultural mulch, while polystyrene (PS) is widely used for food containers and packaging. Microplastics released from these polymers during use and disposal are transported into aquatic systems via urban runoff, wastewater treatment plant effluents, and riverine pathways (Andrady 2011; Kudzin et al. 2023; Waring et al. 2018). Plastics also act as carriers of other toxic compounds by providing surfaces for adsorption (Henry et al. 2018). Therefore, removing them from the environment will reduce both plastic pollution and the spread of attached contaminants.

Research has been conducted on the presence of plastic particles in the environment. However, research on effective removal strategies remains limited. Several physical, chemical, and biological removal methods have been proposed, including filtration, coagulation, advanced oxidation, and microbial degradation, but scale-up and cost-effectiveness remain elusive (Amparán et al. 2025; Kurniawan et al. 2023; Pan et al. 2023). Adsorption has emerged as one of the most practical options because it is easy to apply, inexpensive, and works across many types of pollutants while producing very little secondary waste (Aslam et al. 2025). Among the potential sorbents, clay minerals, particularly montmorillonite (MMT), have drawn attention due to their high surface area, environmental compatibility, and cost-effectiveness (Khan et al. 2025; Sivakumar and Lee 2022; Yang et al. 2018). MMT has been extensively used to remove both organic and inorganic pollutants from the environment. However, its use for capturing MPs and NPs remains largely unexplored. Studies have shown the adsorption of PVC on activated jute stick charcoal and magnetic biochar (Alom et al. 2024; Li and Chen 2024), but to date, no study has investigated the adsorption of PVC on MMT. Recently, evidence has emerged that MMT can adsorb polymeric nanoplastics such as polystyrene (Wang et al. 2024). Building on this gap, our study investigates the interaction between PVC and MMT that could open up new avenues for developing efficient adsorbents that target plastic pollution.

The growing scale of plastic pollution demands that effective adsorbents be developed rapidly. In this context, computational methods offer a valuable tool in investigating potential materials for optimal plastic removal (Townsend 2024). Such methods not only enable rapid screening of candidate adsorbents but also provide molecular-level information about adsorption mechanisms (Azizi et al. 2025; Malloum et al. 2023). Density Functional Theory (DFT) is one of the most well-known computational methods for understanding adsorption at the molecular level. It has been successfully applied to different adsorbate and adsorbent systems. In addition to DFT, molecular dynamics (MD) simulations provide the atomic-scale dynamics and identify the mechanisms governing adsorption over time (Hoang et al. 2024; Sun et al. 2021; Townsend 2024; Yang et al. 2018).

This research employed first-principles methods to explore the atomic and orbital-scale mechanisms underlying PVC adsorption on MMT. Geometry optimization and binding energy calculations were carried out to assess the interaction between adsorbate and adsorbent. Density of states (DOS) and projected DOS (PDOS) were computed to investigate the electronic behavior of the system. A vinyl chloride (VC) trimer was used in the simulation as a representative model for PVC plastic. The trimer preserves the key structural features of the repeating unit and allows simulations to remain computationally feasible. MD simulations, combined with root mean square deviation (RMSD) analysis, were employed to assess the structural stability and dynamic interactions within the VC–MMT hybrid system. To our knowledge, no earlier study has integrated DFT and MD to investigate vinyl chloride adsorption on montmorillonite, and this combined approach provides a more resolved view of the underlying surface interaction.

## Materials and methods

### Computational details

**Fig. 1:**
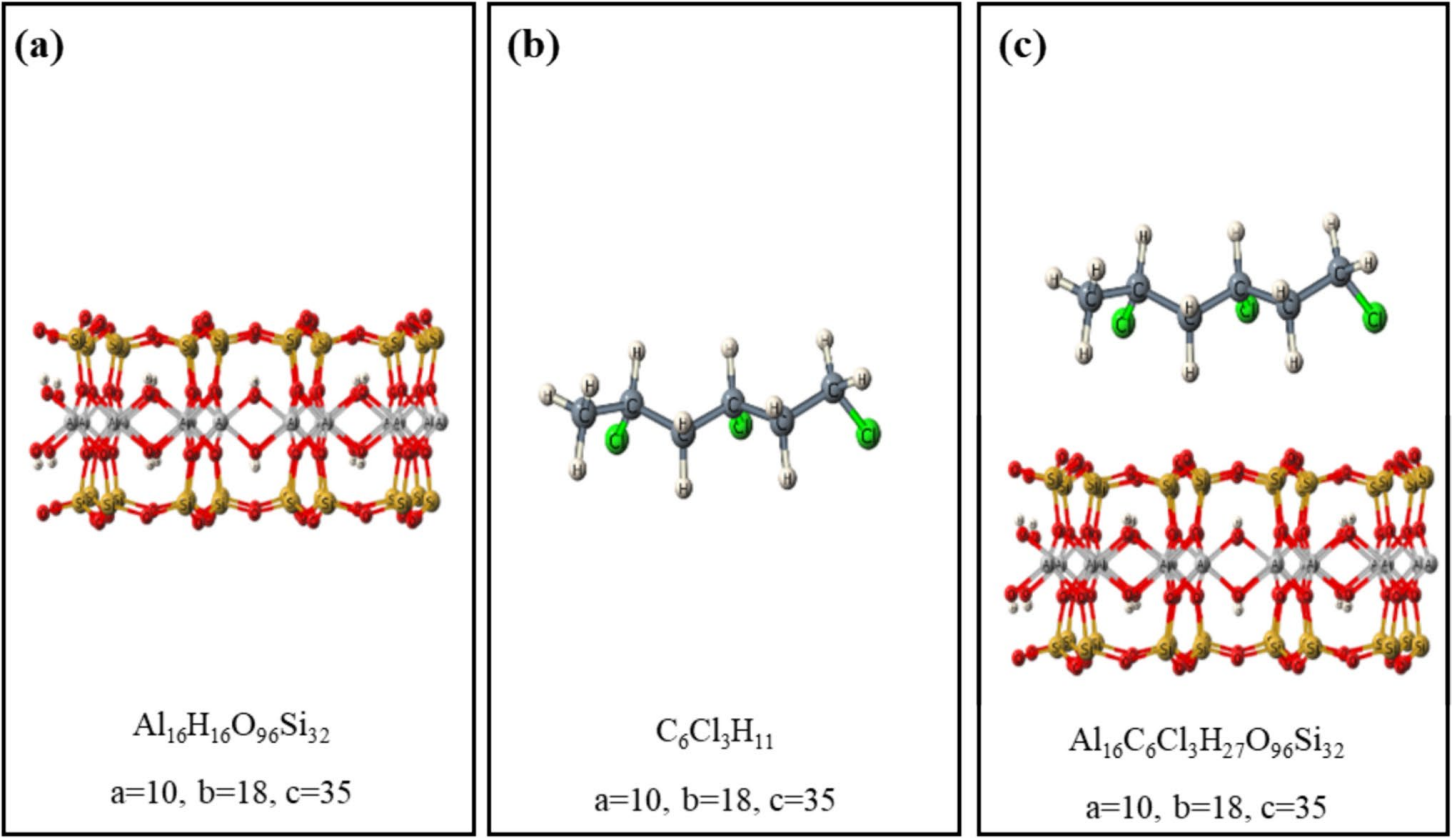
BIOVIA generated models (**a**) MMT (adsorbent). (**b**) VC (adsorbate). (**c**) VC–MMT hybrid. (Red: Oxygen, brown: Silicon, light-gray: Aluminum, white: Hydrogen, green: Chlorine, dark-gray: Carbon)

The models of the study were prepared using Dassault Systèmes, BIOVIA Materials Studio 2020 (Fig. 1). A trimer of vinyl chloride was selected as a simplified model that retains the essential functional groups involved in adsorption while reducing computational cost. Such oligomeric fragments capture the key local electronic properties of the polymer, such as charge distribution, polarization, and surface affinity, which largely control adsorption (Schmid 2022). The MMT used in this work was based on a neutral dioctahedral TOT slab without isomorphic substitutions (see Supplementary Material, Text S1).

The self-consistent DFT equations were solved using the Vienna Ab Initio Simulation Package (VASP). The fictitious one-electron Kohn–Sham (KS) equations were applied with periodic boundary conditions (Eq. (1)). It represents a non-interacting Schrödinger equation that generates the electron density of an interacting particle system (Bechthold et al. 2024; Ghazanfari et al. 2022; Kohn and Sham 1965; Parr 1980).

1 $$\left(\frac{{-\hslash }^{2}}{2m}{\nabla }^{2}+ v\left[\overrightarrow{r}, \rho \left(\overrightarrow{r}\right)\right]\right){\varphi }_{i}^{KS}\left(\overrightarrow{r}\right)= {\varepsilon }_{i}{\varphi }_{i}^{KS}\left(\overrightarrow{r}\right)$$

where $\hslash$ denotes the reduced Planck constant, m is the electron mass, and ∇^2^ is the Laplacian operator. The first term represents the kinetic energy operator. The second term $\mathrm{v}\left[\overrightarrow{\mathrm{r}},\uprho \left(\overrightarrow{\mathrm{r}}\right)\right]$ represents the effective Kohn–Sham potential, which depends on the electron density and includes the external, Hartree, and exchange–correlation contributions. The Kohn–Sham orbitals are shown by ${\varphi }_{i}^{KS}\left(\overrightarrow{r}\right)$ and ${\upvarepsilon }_{\mathrm{i}}$ are the corresponding eigenvalues. The orbitals are integrated with the orbital occupation numbers and used to compute the total electron density.

The KS equation for spin polarization can be written as Eqs. (2) and (3):

2 $$\left(\frac{{-\hslash }^{2}}{2m}{\nabla }^{2}+ {v}_{\alpha }\left[\overrightarrow{r}, {\rho }_{\alpha }\left(\overrightarrow{r}\right)\right]\right){\varphi }_{i\alpha }^{KS}\left(\overrightarrow{r}\right)= {\varepsilon }_{i\alpha }{\varphi }_{i\alpha }^{KS}\left(\overrightarrow{r}\right)$$

3 $$\left(\frac{{-\hslash }^{2}}{2m}{\nabla }^{2}+ {v}_{\beta }\left[\overrightarrow{r}, {\rho }_{\beta }\left(\overrightarrow{r}\right)\right]\right){\varphi }_{i\beta }^{KS}\left(\overrightarrow{r}\right)= {\varepsilon }_{i\beta }{\varphi }_{i\beta }^{KS}\left(\overrightarrow{r}\right)$$

where ${\varphi }_{i\alpha }^{KS}\left(\overrightarrow{r}\right)$ and ${\varphi }_{i\beta }^{KS}\left(\overrightarrow{r}\right)$ are the spin-dependent Kohn–Sham orbitals, and ${\varepsilon }_{i\alpha }$ and ${\varepsilon }_{i\beta }$ are their corresponding eigenvalues. The quantities ${\uprho }_{\alpha }\left(\overrightarrow{\mathrm{r}}\right)$ and ${\uprho }_{\beta }\left(\overrightarrow{\mathrm{r}}\right)$ denote the spin-up and spin-down electron densities. The effective Kohn–Sham potentials are represented by ${v}_{\alpha }$ and ${v}_{\beta }$. The total electron density is obtained from the Kohn–Sham orbitals weighted by their occupation numbers *f*_*i*_ (Eq. (4)).

4 $$\rho \left(\overrightarrow{r}\right)= {\sum }_{i}fi\,{\varphi i}^{KS*}{\varphi i}^{KS}(\overrightarrow{r})$$

The total electron density determines the effective potential in the Kohn–Sham equations. This potential is defined as the functional derivative of the total energy with respect to the electron density. It includes the interaction of electrons with the ionic potential, as well as electron–electron interactions through Coulomb, exchange, and correlation terms. All these equations are solved in an iterative, self-consistent manner by using VASP software. Calculations were performed using the projected augmented wave (PAW) method with Perdew–Burke–Ernzerhof procedure (PBE-functional) (Ghazanfari et al. 2022; Shafei et al. 2023). The Brillouin zone was sampled exclusively at the Γ-point using a Γ-centered k-point scheme (Ghazanfari et al. 2022; Graupner and Kilin 2023). The supercell dimensions were set to 10 Å × 18 Å × 35 Å in the directions of a, b, and c. The plane-wave energy cut-off (ENCUT) was set to 400 eV. This value matched the highest ENMAX among all elements in the system, as specified in the PAW pseudopotentials. The selection ensured a uniform and precise representation of all atomic species in the calculation.

### Binding energy

Binding energy calculation was a central part of this study and was conducted in three sequential steps. First, the individual structures of the adsorbent and adsorbate were geometry optimized. Second, a hybrid system combining both molecules was constructed and optimized. Finally, the total energies of the optimized adsorbent, adsorbate, and hybrid system were used to calculate the binding energy (Eq. (5)) (Heimann et al. 2021), where all energies are expressed in electronvolt (eV).

5 $${\mathrm{E}}_{\mathrm{b}} = {\mathrm{E}}_{hybrid}-({\mathrm{E}}_{adsorbent} + {\mathrm{E}}_{adsorbate})$$

E _hybrid_ is the total energy of the hybrid, while E _*adsorbent*_ represents the energy of MMT and E _*adsorbate*_ is the energy of VC.

### Density of states

The density of states (DOS) describes the number of available electronic states across different energy levels (Bechthold et al. 2024) and was computed using (Eq. (6)).

6 $$n\left(\varepsilon \right)= {\sum }_{i}\updelta (\varepsilon -\varepsilon i)$$

Here, the Dirac delta function is used to count the number of states at a given energy using a finite-width Gaussian function. The DOS reflects the system’s dispersion relation in which higher DOS indicates a greater number of available electronic states. To further explore the role of individual atoms, the Projected Density of States (PDOS) were computed by decomposing the total DOS into atomic contributions, as described in the Supplementary Material (Text S2). The energy difference between the highest occupied molecular orbital (HOMO) and lowest unoccupied molecular orbital (LUMO) is represented as HO–LU gap (Griffith and Orgel 1957). The band gap was calculated to assess the electronic properties of the adsorbent before and after adsorption. Bader charge analysis was performed using charge density from static DFT calculations. The total Bader charge of VC in the hybrid system was obtained by summing the charges of all VC atoms (Eq. (7)). This value was compared with isolated VC to quantify charge redistribution upon adsorption.

7 $$\Delta {Q}_{VC}= {Q}_{VC}^{hybrid}-{Q}_{VC}^{isolated}$$

${Q}_{\mathrm{VC}}$ denotes the Bader charge of VC, with ${Q}_{\mathrm{VC}}^{\mathrm{hybrid}}$ and ${Q}_{\mathrm{VC}}^{\mathrm{isolated}}$ referring to the charges in the hybrid and isolated systems. Charge density difference analysis was performed to visualize electronic redistribution upon VC adsorption on the MMT surface (Eq. (8)).

8 $$\Delta \rho = \rho Hybrid- \rho VC-\rho MMT$$

where ${\rho }_{\mathrm{Hybrid}}$ is the charge density of the VC–MMT system, and ${\rho }_{\mathrm{VC}}$ and ${\rho }_{\mathrm{MMT}}$ are the charge densities of the isolated VC molecule and the isolated MMT surface.

### Molecular dynamics

Molecular dynamics (MD) simulations were performed to track atomic motion over time. The simulations used the Verlet algorithm with a Langevin thermostat in the canonical ensemble (NVT) to maintain constant particle number (N), volume (V), and temperature (T) (Townsend 2024; Tuckerman and Martyna 2000). The temperature was set at 323 K to observe system stability and polymer behavior. A time step of 1 femtosecond (fs) was applied over 1000 steps. Gaussian smearing was used for partial occupancies, and symmetry constraints were removed to allow full atomic movement. The stress tensor was computed while keeping the cell volume and shape fixed. MD trajectories were analyzed to observe structural shifts and assess overall system stability. Root mean square deviation (RMSD) was used to track molecular deformation throughout the simulation (Schreiner et al. 2012). The first frame of the trajectory served as the reference structure, and RMSD values were calculated against all subsequent frames to quantify deviations over time. The VC–MMT interaction energy was monitored as a function of simulation time. Radial distribution functions (RDF) were calculated to analyze spatial correlations between VC atoms and surface oxygen atoms of MMT.

## Results and discussion

### Binding energy

Total energies of each system were analyzed, and binding energy was calculated (Table 1). The binding energy of − 0.62 eV confirms that the adsorption process is exothermic and occurs spontaneously. This energy level aligns with values typically associated with physisorption (Zen et al. 2016) on layered silicates, and in this case sits at the strong end of that range, where the molecules remain held in place by surface-level interactions. The negative value also specifies that the VC–MMT adsorption system is heat-stable (Fang et al. 2022b). A comparable adsorption energy of − 0.38 eV was reported in a study involving carbon dioxide adsorption onto MMT (Wungu et al. 2021). A stronger adsorption was observed (− 2.3 eV), in which MMT was used to adsorb chloro-hydroxypropyl trimethylammonium chloride (Yang et al. 2018). While adsorption studies directly involving PVC are lacking, studies on poly(vinyl alcohol) have demonstrated strong adsorption onto montmorillonite (De Bussetti and Ferreiro 2004). In addition, the synthesis of clay-reinforced PVC composites demonstrates the presence of interfacial interactions between the polymer and the clay minerals (Boraei et al. 2024). Overall, the calculated binding energy confirms that VC adsorption on the MMT surface is energetically favorable and governed by stable, non-covalent interactions.

**Table 1:** Total energies of the systems

Molecule name	Total energy (eV)	Band gap (eV)
Hybrid	− 1279.50	4.0
MMT (adsorbent)	− 1177.02	5.4
VC (adsorbate)	− 101.86	4.6
Binding energy	** − 0.62 eV**	**-**

### Bond length

Interaction between VC and MMT was examined by analyzing interatomic distances. Initially, the VC molecule was placed approximately 3.0 Å above the MMT surface. After structural optimization, two hydrogen–oxygen pairs between the hydrogen atoms of VC and surface oxygen atoms of MMT were identified. In one pair, the distance between H of VC and O of MMT decreased to 1.8 Å, while the other H–O pair was at 2.6 Å. Typically, O–H bonds range from 1.5 Å to 2.0 Å for strong interactions, while weaker interactions can extend up to 2.5 Å to 3.0 Å depending on geometry and partial charge distribution (Benco et al. 2001). Comparable interactions were observed in simulations of pyocyanin on montmorillonite, where methyl hydrogens formed contacts with basal oxygens at distances of about 2.5 Å (Fashina et al. 2024). Such contacts have also been described as unconventional C–H···O interactions (Triptow et al. 2022). Another geometric criterion often used to evaluate surface proximity is interatomic distance relative to the sum of van der Waals radii (Grabowski 2024; Sorroche et al. 2024). The vdW radius is 1.20 Å for H and 1.52 Å for O, totaling 2.72 Å (Chernyshov et al. 2020; Mantina et al. 2009). Both pairs of O–H lie within this vdW radius. These structural features indicate that surface proximity and short-range noncovalent interactions play a key role in stabilizing VC on the MMT surface, supporting a physisorption-dominated interaction mechanism.

### Density of states

**Fig. 2:**
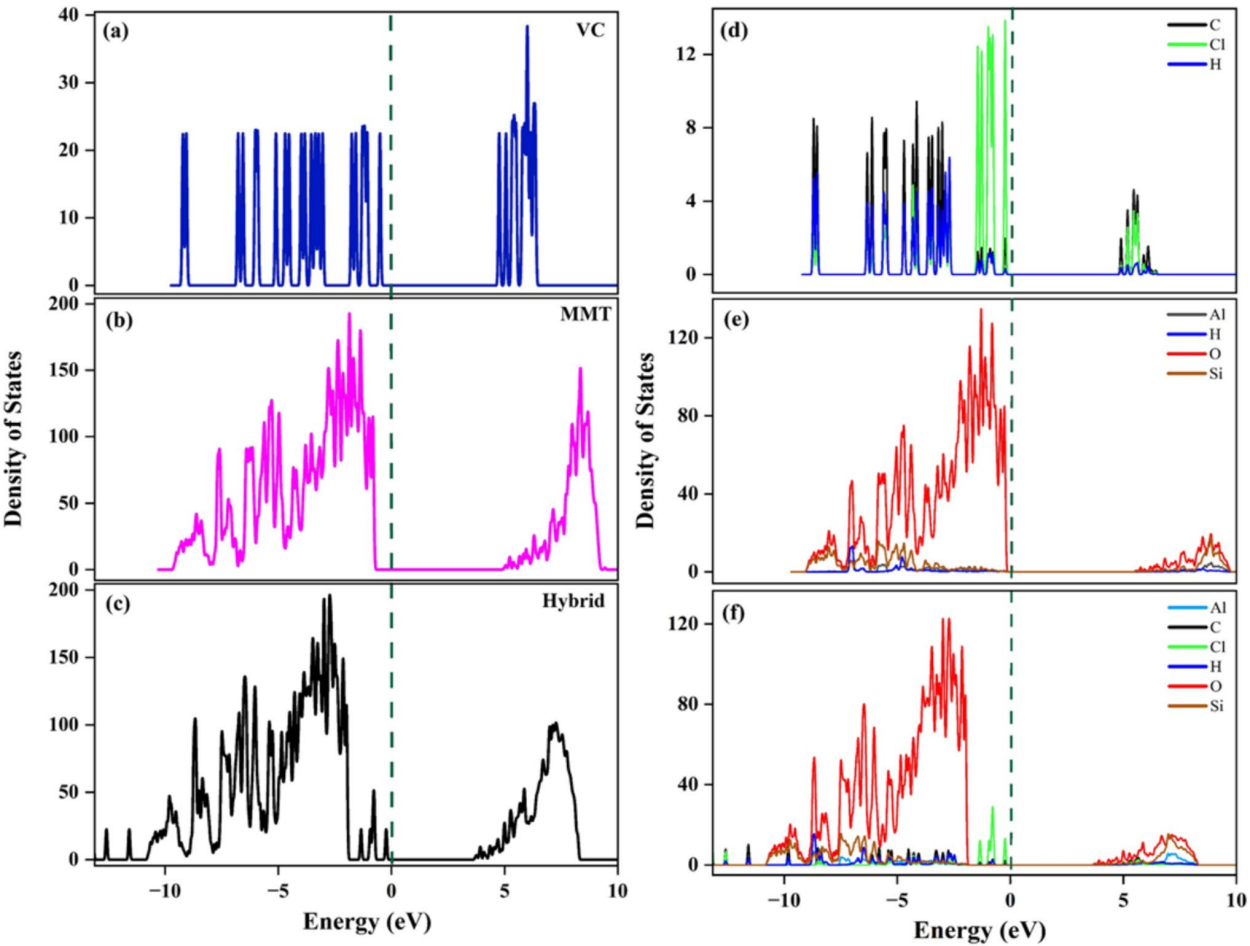
Density of states illustrating the valence and conduction bands for (**a**) VC, (**b**) MMT, and (**c**) the VC–MMT hybrid. The DOS was decomposed (**d**–**f**). The atom-projected total DOS illustrates the overlap of atoms, along with a slight shift of the system toward lower energy after adsorption: (**d**) VC, (**e**) MMT, (**f**) VC–MMT Hybrid

Density of States (DOS) calculations revealed a reduction in the band gap upon adsorption. The band gap decreased from 5.4 eV (MMT) and 4.6 eV (VC) to 4.0 eV in the VC–MMT hybrid (Fig. 2a–c) and indicates modification of the electronic structure due to interaction. These results align with findings from previous studies on MMT (Shafei et al. 2021). A lower band gap generally improves adsorption affinity by facilitating electron exchange between the adsorbate and adsorbent. It also increases the density of electronic states near the Fermi level and facilitates orbital hybridization (Dzade et al. 2014; Liang et al. 2025). Projected DOS was computed for individual atoms within the molecules. It identifies atoms of a system that are involved in electronic interactions and reveals site-specific reactivity and adsorption behavior. In hybrid, PDOS showed noticeable overlap between H and O orbitals from − 10 to − 2 eV (Fig. 2d–f). This overlap indicates interfacial electronic interactions between VC and the MMT surface. Additionally, the hybrid system (Fig. 2c, f) shifted to lower energies (~ − 14 eV) compared to the isolated molecules (~ − 10 eV). This shift likely results from electronic perturbation and polarization induced by adsorption (Bahamon et al. 2021; Fang et al. 2022b).

### PDOS of individual atoms

**Fig. 3:**
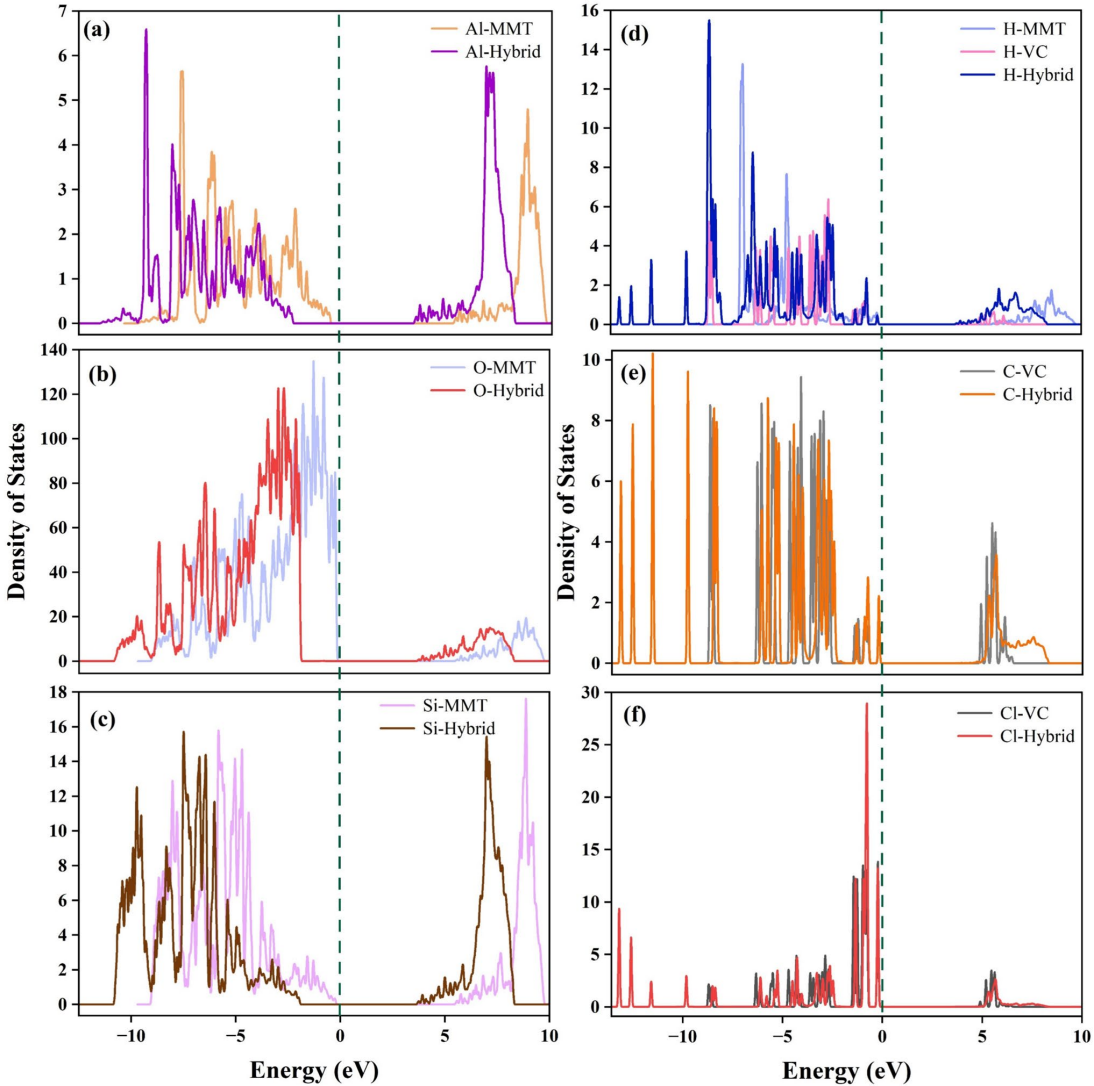
Comparative PDOS analysis of individual atoms (Al, O, Si, H, C, Cl) before and after adsorption of VC on MMT: (**a**–**c**) Al, O, and Si from MMT; (**d**) H from both MMT and VC; (**e**–**f**) C and Cl from VC

The PDOS of individual atom types was extracted to analyze elemental contributions before and after adsorption (Fig. 3). Significant changes in the electronic structure were observed after hybrid formation, which confirms adsorption-induced interactions. Notably, Al, O, and Si exhibited clear shifts toward lower energy states. The valence band (VB) of Al shifted from the range (− 7 to − 2 eV) in the pristine state to (− 9 to − 3.5 eV) after adsorption, while its conduction band (CB) moved from 9 eV to 7.5 eV. Similarly, the VB of Si moved from (− 8 to − 1 eV) to (− 10 to − 3 eV) with its CB shifted from 9 to 7 eV.

In the case of O, VB shifted from (− 8 to − 1 eV) to (− 10 to − 3 eV) and CB shifted from 9 to 7 eV. The overall shift of states toward lower energies indicated a reduction in surface energy, consistent with enhanced stability of the system after adsorption (Fang et al. 2022a). H did not show a significant shift, and C retained its characteristic peaks in the hybrid, while Cl exhibited a minimal shift that indicates weak or negligible orbital overlap. Among the six elements analyzed, O displayed significant DOS near the Fermi level. This suggests its active role in the adsorption process, likely through polarization and orbital interaction with VC. Similar findings were also reported in which CO molecules adsorbed onto Al-embedded graphene (Aravindh et al. 2021; Jiang et al. 2014). In contrast, Al, Si, Cl, and C showed little or no DOS near the Fermi level, suggesting limited direct involvement in adsorption. Thus, the proximity of O electronic states to the Fermi level identifies oxygen as the primary active site facilitating VC adsorption on the MMT surface. Additionally, the changes observed in PDOS after adsorption further indicate improved system stability (Zou et al. 2022). The PDOS shifts observed for Al and Si atoms resulted from indirect electronic effects. Specifically, the adsorption of VC causes electronic redistribution mainly around oxygen atoms that are directly bonded to Al and Si within the MMT structure. This redistribution leads to polarization effects that alter the electronic environments of Al and Si (Fang et al. 2022a; Ferreira et al. 2019).

### Orbital specific PDOS

To understand the orbital contributions to the valence and conduction bands, orbital-specific PDOS was performed (Fig. S1). The interaction primarily involved atom 38 (H) and atom 99 (O) in the hybrid system. These atoms correspond to atom 15 (H) in VC and atom 83 (O) in MMT before adsorption. The analysis revealed that the valence band is predominantly composed of O 2p orbitals that highlight oxygen’s key role in electronic interactions (Dash and Rath 2020). Similar observations were reported previously where occupied states were primarily contributed by O 2p orbitals in kaolinite (Heimann et al. 2021). H contributes primarily through its s orbitals with a noticeable intensity at lower energy levels, indicating localized electronic states (Yan et al. 2024). Specifically, the orbitals of O shifted to lower energy levels that showed a decrease in energy and an increase in the overall stability of the system (Sun et al. 2021). Taken together, the DOS and PDOS results highlight the contribution of surface oxygen states and confirm that adsorption is accompanied by orbital-level interactions without the formation of covalent bonds.

### Charge analysis

**Fig. 4:**
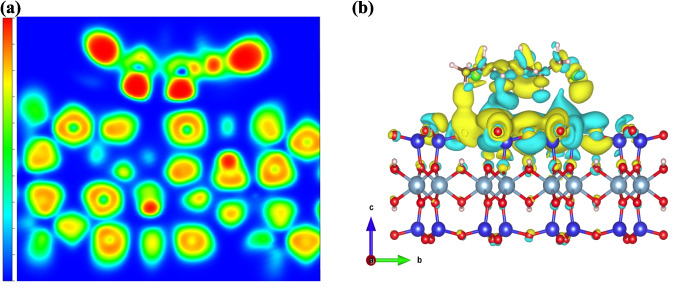
(**a**) Electron localization function (ELF) map for the VC–MMT system, illustrating the spatial distribution of localized electrons at the interface. High ELF values (red/yellow) indicate electron localization around atomic centers, while low ELF values (blue) correspond to delocalized regions. (**b**) Charge density difference map for VC adsorption on the MMT surface; yellow and cyan isosurfaces represent electron accumulation and depletion

Charge analysis was performed to clarify the nature of VC–MMT interaction. The electron localization function (ELF) (Fig. 4a) shows that electrons remain localized around individual atoms. No shared localization region forms between VC and the MMT surface. This indicates the absence of covalent bonding. Bader charge analysis further confirms this behavior. The charge change on the VC molecule after adsorption is approximately 0.012 e, which is negligible. This shows that electron transfer between VC and MMT is minimal and interaction is not driven by a donor–acceptor mechanism. The charge density difference map (Fig. 4b) explains electron accumulation and depletion near surface oxygen atoms and adjacent VC regions. No continuous charge density is observed across the interface. Based on the charge analysis, VC adsorption on the MMT surface occurs through multiple weak interactions. Van der Waals forces provide the primary attractive interaction. Electrostatic polarization arises from the oxygen-rich surface of MMT. Weak and transient hydrogen bonding may occur locally between VC hydrogens and surface oxygen atoms.

This mechanistic study does not generate adsorption isotherms. However, the nonuniform and oxygen-rich sites of montmorillonite suggest adsorption on a heterogeneous surface. Freundlich or hybrid Sips models may therefore be more suitable than a purely Langmuir framework. Future experimental validation using these models can clarify the role of surface heterogeneity in VC adsorption.

### Molecular dynamics

**Fig. 5:**
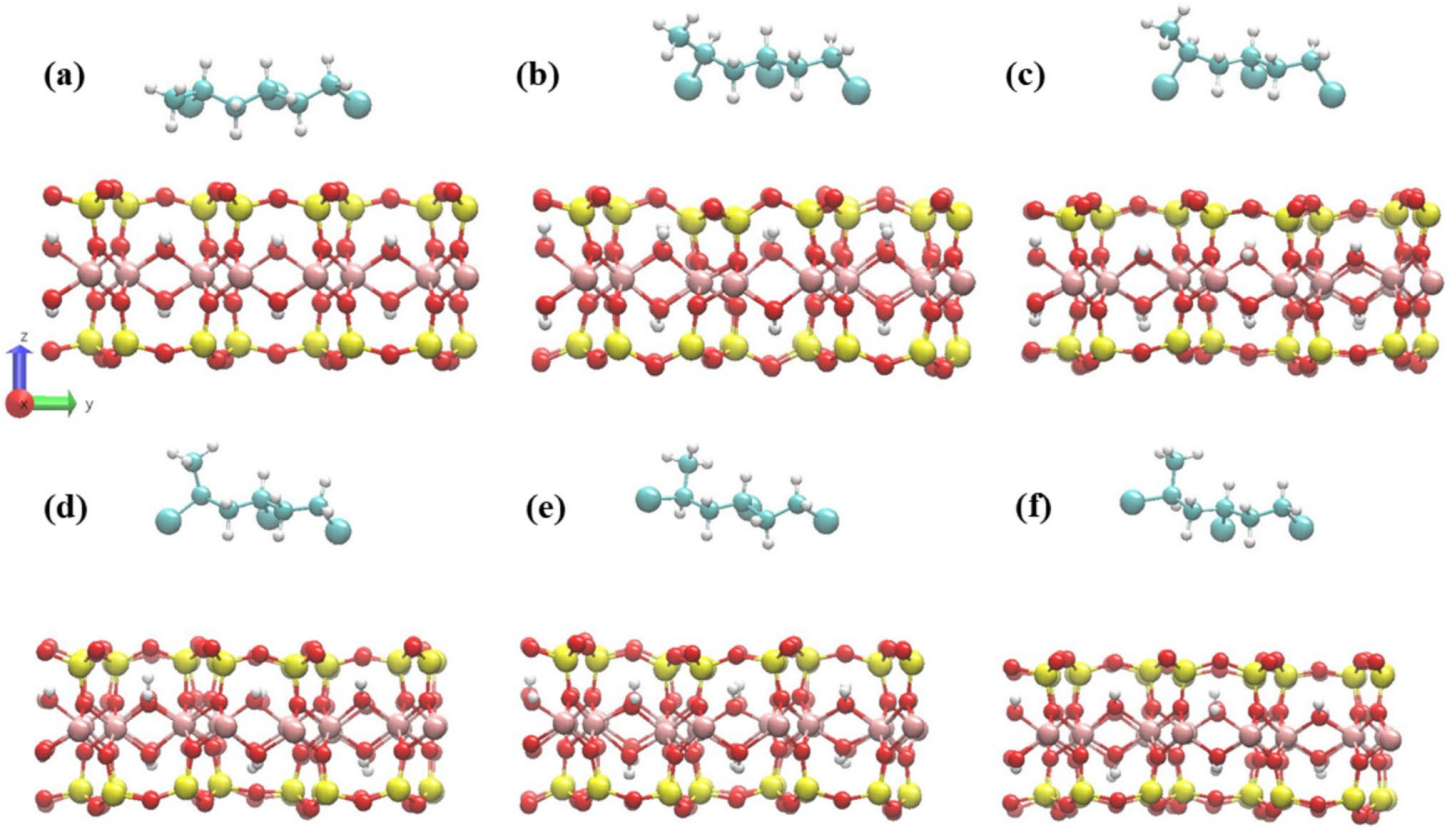
MD simulation of VC with MMT, computed with 1000 time steps; at (**a**) 0, (**b**) 200, (**c**) 400, (**d**) 600, (**e**) 800, and (**f**) 1000 fs

Molecular dynamics (MD) simulations were performed to analyze the interaction behavior between VC and MMT. The MMT structure remained stable throughout the simulation, and its atoms exhibited localized vibrations that indicated thermal motion at fixed lattice positions. This confirms the structural integrity of MMT under simulated conditions (Fig. 5), consistent with earlier clay-based studies (Ghazanfari et al. 2023; Qu et al. 2023). In contrast, significant atomic motion was observed for the VC molecule. The central region of VC remained oriented toward the MMT surface during the simulation. Atoms near the edge of VC showed positional fluctuations over time. This behavior indicates surface association without rigid bonding.

**Fig. 6:**
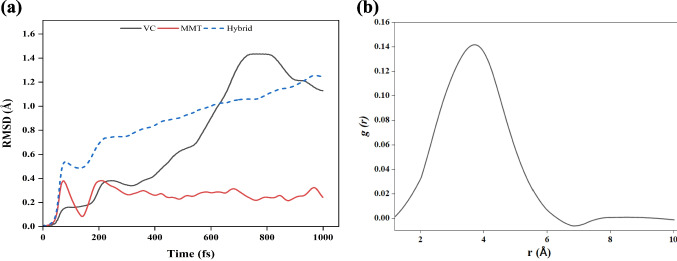
(**a**) RMSD profiles of VC, MMT, and the VC-MMT hybrid system over 1000 frames. (**b**) Radial distribution function $g(r)$ between VC atoms and surface oxygen atoms of the MMT obtained from molecular dynamics simulations

Root mean square deviation (RMSD) analysis was conducted to assess the structural stability of VC, MMT, and their hybrid system during MD simulation (Fig. 6a). The RMSD of MMT remained low throughout the 1000 fs simulation and fluctuated within a narrow range of 0.2 Å to 0.3 Å. This indicates structural rigidity and confirms the stability of the layered silicate framework (Ghazanfari et al. 2023). In contrast, VC showed a gradual increase in RMSD, reaching about 1.5 Å at 750 fs, followed by a slight decrease toward the end of the simulation (1000 fs). This behavior reflects the flexibility of VC and indicates molecular reorientation and fluctuation driven by weak, non-covalent surface interactions (Schreiner et al. 2012). The hybrid system showed an RMSD trajectory that increased gradually and reached around 1.3 Å at the end of the simulation. This trend is mainly governed by the motion of VC while MMT remained nearly unchanged. The absence of abrupt spikes in the RMSD trajectory of the hybrid system confirms that structural integrity was maintained under simulated conditions. Similar RMSD values were reported by others from MD simulation of 2, 3-dihydroxypropanal on MMT (Vojood et al. 2022).

To further assess the stability of the VC–MMT interaction during simulation, the interaction energy was monitored as a function of time (Fig. S2). After an initial equilibration period, the interaction energy fluctuates around a stable mean. The absence of systematic energy drift indicates that VC remains associated with the MMT surface without forming strong chemical bonds. The corresponding RDF (Fig. 6b) displays a broad first peak at relatively long distances, consistent with time-averaged thermal motion and the absence of strong, specific interactions. In this study, simulations were performed in a vacuum to isolate the intrinsic interaction between VC and the MMT surface and to establish a clear mechanistic baseline. This approach enables examination of fundamental adsorption behavior without interference from solvent-induced effects. Future studies incorporating explicit water molecules can be built on this foundation to assess adsorption under aqueous conditions.

## Conclusions

Simulations conducted within this research establish DFT and MD as effective tools for selecting adsorbents for specific pollutants (plastics in this case). This study demonstrates montmorillonite (MMT) as a suitable adsorbent for capturing vinyl chloride (VC), a representative microplastic pollutant. DFT calculations performed using VASP provided detailed insights into the adsorption mechanism. The negative binding energy (− 0.62 eV) confirms favorable and spontaneous interactions between VC and MMT. Electronic structure analyses revealed a reduction in the HOMO–LUMO band gap from 5.4 eV (MMT alone) to 4.0 eV (VC–MMT hybrid), reflecting interfacial electronic perturbation upon adsorption. Projected Density of States analysis identified the dominant role of surface oxygen states, with contributions from O 2p orbitals of MMT and H 1 s orbitals of VC, indicating orbital-level interactions. Charge analysis, including ELF, Bader charge, and charge density difference mapping, confirms negligible electron transfer and the absence of covalent bonding. Adsorption is governed by polarization-driven physisorption supported by van der Waals interactions. MD simulations further supported this mechanism by demonstrating stable surface association of VC with MMT over the simulated time window. The interaction energy remains stable, RMSD analysis confirms the structural integrity of the hybrid system, and RDF analysis indicates weak, non-specific contacts rather than persistent hydrogen bonding. Collectively, these results confirm that VC adsorption onto MMT is energetically favorable, structurally stable, and driven by noncovalent surface interactions. These findings highlight montmorillonite as a realistic and robust sink for PVC-related species in environmental systems and provide a molecular-level framework for understanding microplastic–clay interactions.

## Supplementary Information

Below is the link to the electronic supplementary material.

ESM 1 (DOCX 245 KB)

## Data Availability

Data will be made available on request.
