# Supplementary material for: Mechanistic insights into PVC microplastic adsorption on montmorillonite: A first-principles approach toward pollution control
Source: Environ Sci Pollut Res Int. 2026 Feb 5;33(7):2936–48. doi: 10.1007/s11356-026-37449-w (PMC12982257; doi:10.1007/s11356-026-37449-w)
Supplement: Supplementary file 1 — (DOCX 245 KB) [file 11356_2026_37449_MOESM1_ESM.docx]

**Supplementary Material**

**Mechanistic Insights into PVC Microplastic Adsorption on Montmorillonite: A First-Principles Approach Toward Pollution Control**

Hafiz Muhammad Umer Aslam ^1,2,4^, Achintya Bezbaruah^1,2*^, Dmitri Kilin^3^

^1^Civil, Construction and Environmental Engineering, North Dakota State University, Fargo, ND 58102, USA

^2^ Environmental and Conservation Sciences, North Dakota State University, Fargo, ND 58102, USA

^3^ Chemistry and Biochemistry, North Dakota State University, Fargo, ND 58102, USA

^4^ Department of Chemistry, School of Science, University of Management and Technology, Lahore, 54770, Pakistan

Achintya Bezbaruah ([a.bezbaruah@ndsu.edu](mailto:a.bezbaruah@ndsu.edu))

**Text S1:**

A dioctahedral 2:1 phyllosilicate slab was used as the reference montmorillonite surface model. The layer was built without explicit isomorphic substitutions or interlayer charge-balancing ions, which is a common computational approach when the primary objective is to isolate intrinsic surface–adsorbate interactions. This configuration permits direct examination of PVC contact with the siloxane surface before the added complexity of interlayer cations, hydration, and charge heterogeneity is introduced in future simulations.

**Text S2:**

PDOS calculations were performed on fully optimized structures using static runs with an electronic convergence threshold of 1×10^-6^ eV. Gaussian smearing with a width of 0.05 eV was applied, and the DOS was sampled from –15 to +10 eV over 3000 points. Site- and orbital-resolved contributions were obtained using LORBIT = 11 with atom-specific projection radii. PDOS of VC and MMT were resolved by element types (C, Cl, H) and (Al, H, O, Si), respectively. For MMT, surface oxygen atoms primarily contribute through their O 2p orbitals, while Si and Al atoms contribute mainly via Si 3p and Al 3p states. The PDOS of the hybrid contained all these elements to capture the combined electronic characteristics. The atomic-level interactions were elucidated by decomposing PDOS of the bonded atoms into their orbital contributions (s, p, d and f). The involvement of different orbitals is crucial for understanding the nature of the adsorbate-adsorbent interaction.

|  |
| --- |
|  |

| 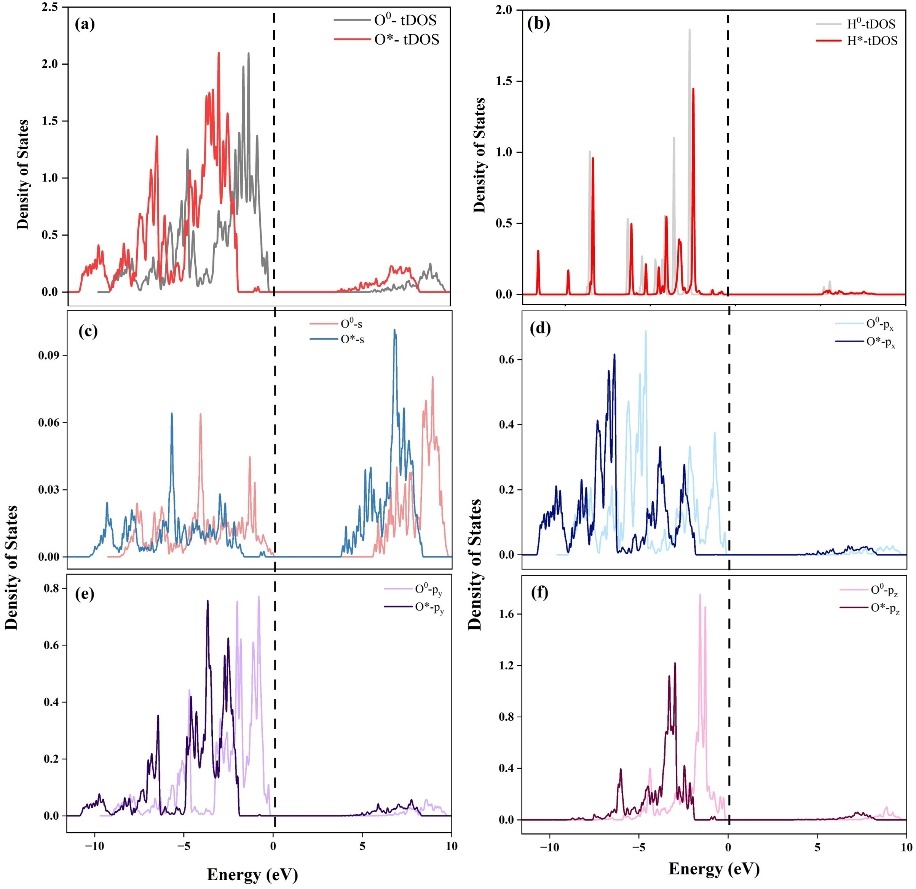 |
| --- |
| **Fig. S1** Comparison of atomic orbitals before and after adsorption of VC on MMT to assess their involvement in the adsorption process. Atoms 15 (H) from VC and 83 (O) from MMT, were compared with the corresponding atoms 38 (H) and 99 (O) in the hybrid system (after adsorption). (a-b) Total density of states of O and H. (c-f) orbitals of the O atom |

| 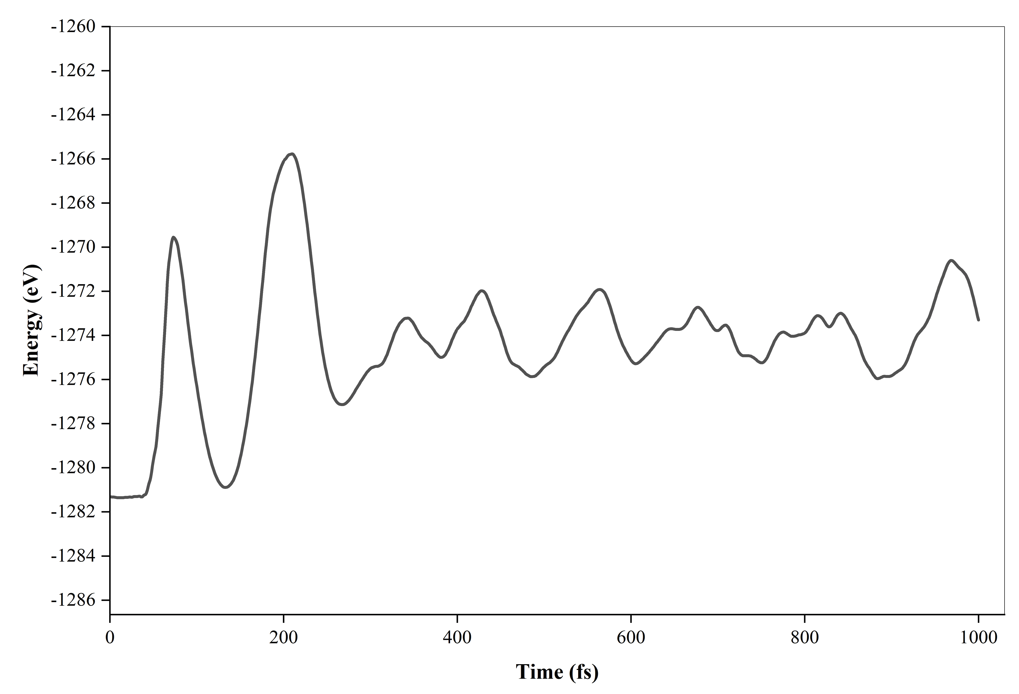 |
| --- |
| **Fig. S2** Time evolution of the VC–MMT interaction energy during molecular dynamics simulation, showing initial equilibration followed by stable fluctuations, indicative of sustained interfacial association over the simulated time window. |
